# Linking white matter hyperintensities to regional cortical thinning, amyloid deposition, and synaptic density loss in Alzheimer's disease

**DOI:** 10.1002/alz.13845

**Published:** 2024-04-22

**Authors:** Junfang Zhang, Haijuan Chen, Jie Wang, Qi Huang, Xiaomeng Xu, Wenjing Wang, Wei Xu, Yihui Guan, Jun Liu, Joanna M Wardlaw, Yulei Deng, Fang Xie, Binyin Li

**Affiliations:** ^1^ Department of Neurology & Institute of Neurology Ruijin Hospital affiliated with Shanghai Jiao Tong University School of Medicine Shanghai China; ^2^ Clinical Neuroscience Center Ruijin Hospital LuWan Branch Shanghai Jiao Tong University School of Medicine Shanghai China; ^3^ PET Center Huashan Hospital Fudan University Shanghai China; ^4^ Centre for Clinical Brain Sciences University of Edinburgh Edinburgh UK; ^5^ UK Dementia Research Institute University of Edinburgh Edinburgh UK

**Keywords:** Alzheimer’s disease, cortical thickness, MRI, PET, synaptic density, white matter hyperintensities

## Abstract

**INTRODUCTION:**

We investigated the association between white matter hyperintensities (WMH) and regional cortical thickness, amyloid and tau deposition, and synaptic density in the WMH‐connected cortex using multimodal images.

**METHODS:**

We included 107 participants (59 with Alzheimer's disease [AD]; 27 with mild cognitive impairment; 21 cognitively normal controls) with amyloid beta (Aβ) positivity on amyloid positron emission tomography (PET). The cortex connected to WMH was identified using probabilistic tractography.

**RESULTS:**

We found that WMH connected to the cortex with more severe regional degeneration as measured by cortical thickness, Aβ and tau deposition, and synaptic vesicle glycoprotein 2 A (SV2A) density using ^18^F‐SynVesT‐1 PET. In addition, higher ratios of Aβ in the deep WMH‐connected versus WMH‐unconnected cortex were significantly related to lower cognitive scores. Last, the cortical thickness of WMH‐connected cortex reduced more than WMH‐unconnected cortex over 12 months.

**DISCUSSION:**

Our results suggest that WMH may be associated with AD‐intrinsic processes of degeneration, in addition to vascular mechanisms.

**Highlights:**

- We studied white matter hyperintensities (WMHs) and WMH‐connected cortical changes.
- WMHs are associated with more severe regional cortical degeneration.
- Findings suggest WMHs may be associated with Alzheimer's disease–intrinsic processes of degeneration.

## 1 BACKGROUND

White matter hyperintensities (WMHs) visible on T2‐weighted magnetic resonance imaging (MRI) are frequently observed in older individuals.^1^ WMHs are considered neuroimaging markers of cerebral small vessel disease (SVD),^2^ but are also related to Alzheimer's disease (AD).^3^ Previous pathological studies reported an association between WMHs and AD pathologies, indicating the possibility that degenerative pathologies contribute to WMHs, in addition to vascular disease.^4^

WMHs are reported to contribute to the thinning of the regional cortex connected to WMHs in SVD patients.^5^, ^6^, ^7^, ^8^ Previous studies demonstrated that the microstructural metrics of white matter tracts passing through WMHs were related to regional cortical abnormalities in the WMH‐connected cortex as measured by cortical thickness, R1, R2*, and susceptibility values, and further impaired processing speed in SVD.^6^ The relationship between WMHs and cortical changes in WMH‐connected cortex in AD remains unclear. In addition to the possible findings of cortical thinning, we hypothesize that WMHs may correlate with amyloid beta (Aβ) and tau deposition, as well as synaptic density change, in WMH‐connected cortex.

Positron emission tomography (PET) imaging with different tracers can help to detect cortical accumulation of amyloid Aβ plaques and neurofibrillary tau tangles.^9^, ^10^ Recent advances of synaptic PET imaging, with ^18^F‐SynVesT‐1 targeting synaptic vesicle glycoprotein 2 A (SV2A), provides a direct measure of synaptic density in human brain in vivo.^11^ Therefore, combining the amyloid PET, tau PET, and SV2A PET, we can assess the changes in Aβ, tau, and synaptic density in WMH‐connected cortex.

Previous research has shown that WMHs relate to cognitive impairment, but WMH load correlates inconsistently with cognitive function.^12^ This clinico‐radiographic mismatch may be partially explained by the location‐specific effects of WMHs on different cognitive domains.^13^, ^14^, ^15^, ^16^ Histopathologic studies reported that smooth periventricular WMHs (pWMHs) and punctate deep WMHs (dWMHs) are associated with mild changes of the brain parenchyma, whereas irregular pWMHs and confluent dWMHs are associated with more severe parenchymal changes, including damage to myelin and incomplete parenchymal destruction.^17^ Different locations of WMHs may reflect different underlying disease processes, which are complicated by the coexistence of both AD and SVD pathologies in older people.^18^

The primary goal of this study is to assess the correlation of WMHs with cortical changes in WMH‐connected cortex using multimodal neuroimaging data in participants within the AD continuum. We hypothesized that the cortical metrics of thickness, Aβ, tau, and synaptic density are different between WMH‐connected cortex and the corresponding unconnected cortex. To achieve this goal, we will use the ratio as a means of comparing metrics in WMH‐connected cortex and WMH‐unconnected cortex. A ratio of 1 signifies that the two types of cortices have similar parameters. We focus on the ratios that are either > 1 or < 1. We used several neuroimaging modalities, including structural and diffusion MRI, florbetapir (AV45) amyloid PET, ^18^F‐MK‐6240 tau PET, and SV2A PET to measure the cortical thickness, Aβ, tau, and synaptic density. We investigated the possible location‐dependent effects of WMHs on cortical abnormalities by classifying WMHs into pWMHs and dWMHs. We further explored the WMH pattern by dividing white matter into different anatomical brain regions, which included four cortical lobar regions (frontal, temporal, parietal, and occipital lobes). We linked the relevant cortical measures to cognitive function and evaluated the longitudinal effects of WMHs on cortical thinning in WMH‐connected cortex in AD.

## 2 METHOD

The study was approved by the ethics committee, Ruijin Hospital, Shanghai Jiao Tong University School of Medicine, China. All participants in the study or their caregivers signed written informed consent after fully understanding the procedure involved. We certify that the study was conducted in accordance with the ethical standards of the 1964 Declaration of Helsinki and its later amendments. The study was registered with ClinicalTrials.gov (NCT05623124).

### 2.1 Participants and study design

Data were derived from the Ruijin Neurobank of Alzheimer's Disease and Dementia (RJNB‐AD) cohort study, which is an ongoing prospective, observational study with emphasis on neuroimaging to improve the understanding of risk factors, causes, and clinical consequences of AD. The criteria for AD and mild cognitive impairment (MCI) followed the research criteria proposed by the National Institute on Aging–Alzheimer's Association (NIA‐AA, 2011) workgroups, including both clinical features and one biomarker,^19^ based on cognitive status, neuropsychologic performance, and Aβ positivity on amyloid PET determined by agreement between nuclear medicine specialists and one memory‐disorder specialist.

**FIGURE 1 alz13845-fig-0001:**
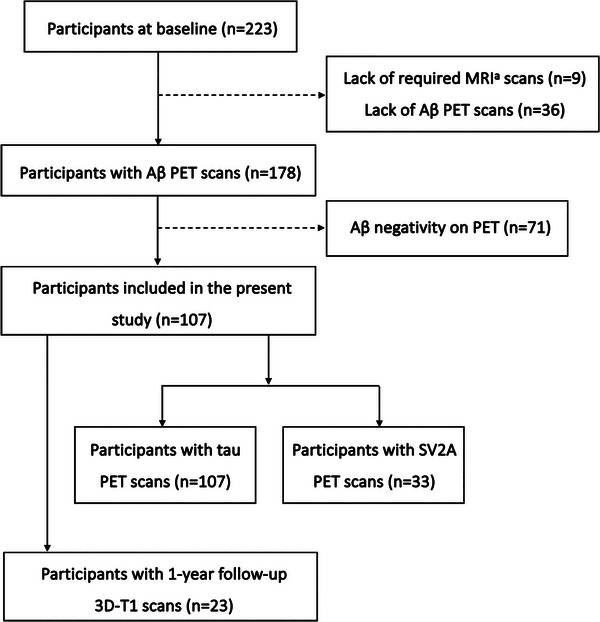
Flow chart of the present study. ^a^The required MRI scans included T1, FLAIR, and multishell diffusion MRI sequences. Aβ, amyloid beta; FLAIR, fluid attenuated inversion recovery; MRI, magnetic resonance imaging; PET, positron emission tomography; SV2A, synaptic vesicle glycoprotein 2 A

For the cross‐sectional analysis, we included 107 participants who tested positive for Aβ on amyloid PET, based on the availability and sufficient quality of T1, fluid‐attenuated inversion recovery (FLAIR), multishell diffusion MRI (dMRI), and amyloid PET scans (Figure 1). At the end of April 2023, 23 out of 107 participants had 1‐year follow‐up MRI data.

All participants were assessed by Mini‐Mental State Examination (MMSE, Chinese Version),^20^ global Clinical Dementia Rating (CDR), 20‐minute Auditory Verbal Learning Test (AVLT), Boston Naming Test (BNT), Shape‐Trail Test (STT‐A and STT‐B), and Animal Fluency Test (AFT).

The participants were categorized into three groups based on their cognitive performance: 59 AD patients (CDR > 0.5), 27 with MCI (CDR = 0.5), and 21 cognitively normal controls (CN, CDR = 0).

### 2.2 Acquisition of MRI and PET images

MRI data were acquired on a 3T scanner (uMR 890, United Imaging Healthcare: https://www.united‐imaging.com/zh‐cn/product‐service/products/mr/umr‐890) with a dedicated 64‐channel head coil. We collected three‐dimensional (3D) T1‐weighted, 3D FLAIR and multishell diffusion MRI images. Detailed parameters for each sequence are provided in the supporting information.

The PET scans were collected on a 3T whole‐body PET/MR scanner (uPMR 790, United Imaging). The participants received an intravenous injection of ^18^F florbetapir (AV45) at a mean dose of 3.7 MBq/kg body weight to image Aβ. Static AV45‐PET data were acquired in sinogram mode 50 minutes after injection. All participants also underwent static ^18^F MK‐6240 PET scan to image tau. In addition, 33 participants had SV2A PET scans to image synaptic density as previously described.^21^ Among these participants, 18 received a diagnosis of AD, 9 were diagnosed with MCI, and the remaining 6 participants were classified as CN. In brief, participants did not take drugs targeted to SV2A for at least 24 hours before PET scan. A 30‐minute static PET scan started at 60 minutes post injection of ^18^F‐SynVesT‐1 (≈ 3.7 MBq/kg body weight). The scans were scheduled at least 1 week apart from each other.

**Research in context**

1. **Systematic Review**: The authors reviewed the literature using traditional (e.g., PubMed) sources. Although the reports of white matter hyperintensities (WMHs) relating to regional cortical changes in Alzheimer's disease (AD) were not exhaustive, the relevant citations are cited appropriately.
2. **Interpretation**: Our findings suggest that WMHs are likely to be associated with AD‐intrinsic processes of degeneration, in addition to vascular mechanisms.
3. **Future Directions**: Given the link between WMHs and regional cortical changes, the effect of WMH change on changes in regional cortex should be assessed longitudinally in future research.

### 2.3 WMH segmentation

**FIGURE 2:**
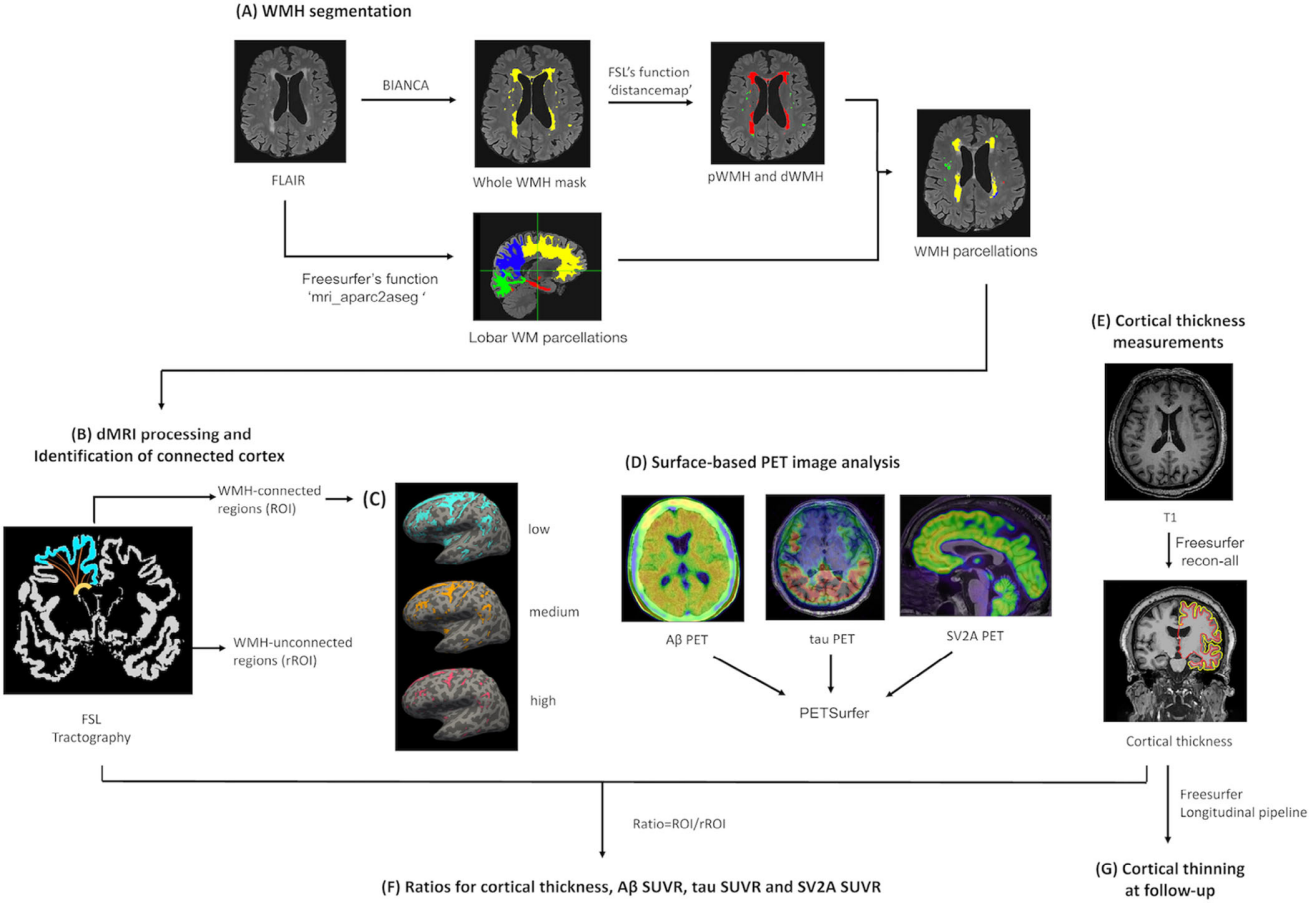
Methodological approach of the performed neuroimaging analysis. A, WMHs were automatically segmented with a k‐nearest neighbor algorithm, and were divided into pWMHs and dWMHs by a 10 mm distance threshold from the ventricles. The pWMHs and dWMHs were further divided according to the lobar WM parcellations. B, Probabilistic tractography was performed from WMHs (yellow color) to the white/gray matter boundary to determine the WMH‐connected cortex (blue color), the WMH‐unconnected cortex (white color) and the connecting white matter tracts (orange color); the cortical terminations of the connecting white matter tracts were projected to the surface. C, Example images of cortical ROIs at different probabilities of connectivity with WMH. D, Aβ PET, tau PET, and SV2A PET were processed using PETsurfer with surface‐based analysis. E, Cortical thickness was measured with FreeSurfer pipeline. F, The cortical metrics of ROI were divided by the corresponding cortical metrics of rROI, resulting ratios for each cortical metric. G, Changes in cortical thickness over time were measured using FreeSurfer longitudinal stream. Aβ, amyloid beta; BIANCA, Brain Intensity AbNormality Classification Algorithm; dMRI, diffusion magnetic resonance imaging; dWMHs, deep white matter hyperintensities; FSL, the FMRIB Software Library; PET, positron emission tomography; pWMHs, periventricular white matter hyperintensities; ROI, region of interest; rROI, reference region of interest; SUVR, standardized uptake value ratio; SV2A, synaptic vesicle glycoprotein 2 A; WM, white matter; WMHs, white matter hyperintensities

We used[Fig alz13845-fig-0001] FSL's (version 6.0, https://fsl.fmrib.ox.ac.uk/fsl/) Brain Intensity AbNormality Classification Algorithm (BIANCA), a fully automated, supervised k‐nearest neighbor (k‐NN) algorithm for WMH segmentation (Figure 2A).^22^ The total hyperintensities’ binary masks were carefully inspected and manually corrected to generate the WMH binary masks. Derived masks of WMH were divided into pWMH and dWMH by defining a 10 mm distance threshold from the ventricles.^23^ Additionally, white matter was labeled according to frontal, parietal, temporal, and occipital lobe parcellations obtained with FreeSurfer's pipeline, which were projected onto the white matter using the “mri_aparc2aseg” function. Using these lobar masks, WMH segmentations were further parcellated into four lobar regions (Figure 2A). Finally, the WMHs were segmented into eight parcels (pWMHs and dWMHs with four lobar regions each). All maps were visually inspected and manually corrected as necessary.

### 2.4 Diffusion MRI processing and identification of connected cortex

The original diffusion MRI data were corrected for susceptibility‐induced distortion by the top‐up method,^24^ and then corrected for head motion and eddy currents using the eddy tool in FSL (version 6.0).^25^ Probabilistic tractography was performed using the probtractx2 function in FSL with the default parameters.^26^ WMH mask was used as the seed mask and the white matter (WM) /gray matter (GM) boundary derived from FreeSurfer (version 7.1.1) as the target mask, generating the “fdt_path” images. In the cortical surface of the “fdt_path” image, each voxel value represents the count of streamlines that connect the cortex and WMHs. The voxel values in the cortical part of the “fdt_path” image were normalized by dividing the total number of streamlines sent out from the seed masks (5000 times per voxel), producing a probability map of the cortex connected to WMHs (Figure 2B). This probability map was further thresholded at three levels (low, medium, and high; Figure 2C). Although lower levels are more susceptible to noise and may lead to false‐positive tracts, higher levels are more specific but might eliminate subordinate pathways.^27^

Cortical areas with a low probability of connectivity were determined using the threshold of the lowest level at 3.08 × 10^−5^ percent of the total streamlines sent out from the seed masks, based on previous literature.^6^, ^28^ Cortical areas with medium and high probability of connectivity were determined by gradually increasing the number of tractography samples until the resulting surface size reached 50% (medium probability) or 25% (high probability) of the initial (low probability) surface size.^27^ The cortical area connected with the WMHs was defined as the cortical region of interest (ROI). Cortex outside the ROI (WMH‐unconnected cortex) was defined as reference ROI (rROI).

### 2.5 Cortical thickness measurements

The 3D T1 image was fed into the standard “recon‐all” processing pipeline within FreeSurfer to reconstruct the cortical surface and estimate cortical thickness (Figure 2E). The resulting cerebrospinal fluid (CSF)/GM boundary (pial surface) and GM/WM boundary (WM surface) were visually inspected and manually corrected as necessary to avoid obvious reconstruction errors. Cortical thickness was measured as the distance between the pial surface and the WM surface.

Considering the non‐uniform distribution of cortical thickness across the brain regions, it will be hard to attribute any potential cortical thinning in WMH‐connected regions to the remote effects of WMH or the inter‐regional differences in cortical thickness.^6^ To minimize this effect, we split the ROIs and rROIs into six cortical regions, including frontal, temporal, parietal, occipital, cingulate, and insula cortex. The mean cortical thickness of ROI was divided by the mean cortical thickness of rROI within each region, resulting in six cortical thickness ratios of ROI to rROI. Subsequently, the averaged cortical thickness ratio was calculated by taking the mean of the six cortical thickness ratios obtained from the previous step (Figure 2F).

### 2.6 Surface‐based PET image analysis

An automatic pipeline was used to extract cortical standardized uptake value ratios (SUVR) by PETSurfer in FreeSurfer (version 7.1.1; https://surfer.nmr.mgh.harvard.edu/fswiki/PetSurfer) using the cerebellum cortex as the reference region (Figure 2D). In detail, structural T1 images were used to create a high‐resolution segmentation using the Schaefer function atlas^29^ to run the partial volume correction (PVC) methods. The PET/anatomical image registration was then performed and visually checked. To minimize the partial volume effect from cortical atrophy in AD, the extended Muller–Gartner method as a PVC method^30^, ^31^ was applied. Surface‐based Aβ map, tau map, and SV2A map were smoothed on the two‐dimensional surface by a Gaussian kernel of 5 mm in full width at half maximum (FWHM). The mean SUVR of ROI was divided by the mean SUVR of rROI within six cortical regions, including frontal, temporal, parietal, occipital, cingulate, and insula cortex, resulting in six SUVR ratios of ROI to rROI. Subsequently, the averaged SUVR ratio was calculated for each map by taking the mean of the six SUVR ratios obtained from the previous step (Figure 2F).

### 2.7 Cortical thinning at follow‐up

We used an automatic longitudinal pipeline^32^ within FreeSurfer to extract estimates for the change in cortical thickness between baseline and 1‐year follow‐up 3D T1 images (Figure 2G). The procedure is optimized for longitudinal scans using an unbiased within‐subject template space and image.^33^ Processing steps are initialized with common information from this template. The longitudinal pipeline within FreeSurfer provides significantly higher statistical power than the cross‐sectional processing.^32^ The cortical segmentations were carefully inspected and manually corrected as necessary. We estimated the cortical thickness at 12 months using the WMH‐connected and unconnected ROIs identified at baseline.

### 2.8 Statistical analyses

Proportions were used to describe the categorical variables; means with standard deviation (SD) or median with the interquartile range (IQR) were used for continuous variables according to their distribution. The group differences between AD, MCI, and CN were estimated using analysis of variance (ANOVA) for continuous variables. To assess the cortical abnormalities in the WMH‐connected cortex, the cortical thickness ratio and SUVR ratios were compared to a value of 1 using one‐sample *t* tests. Normality was assessed using the Shapiro–Wilk test, as presented in Table S1 in supporting information. In cases in which the ratios were not normally distributed, the Mann–Whitney *U* test was used in place of the *t* test, and the Kruskal–Wallis *H* test was used as the non‐parametric equivalent of ANOVA.

To examine the relationship between cortical abnormalities in the WMH‐connected cortex and cognitive performance, we used linear regression models. The ratio of the WMH‐connected cortex to the corresponding WMH‐unconnected cortex for each cortical metric at each connectivity level was used as the independent variable, while cognitive performance served as the dependent variable. We adjusted for age, years of education, and WMH volumes in the regression models.

For the analysis of the longitudinal data, we used repeated‐measures ANOVA to assess the time‐dependent changes in the cortical thickness ratio. To ensure comparability on a consistent scale, the baseline MMSE scores and ratio values were standardized by subtracting their respective means and dividing by their individual standard deviations. The mean and SD of both measures at baseline were used to standardize their follow‐up values at 12 months. Following standardized preprocessing, to directly compare the rate of change between the ratio and MMSE within 12 months, we used a linear mixed model to compare the time‐dependent slope of cognition and cortical thickness ratio.

All statistical analyses were conducted using Python with the Pingouin package^34^ (version 0.5.3). The significance level was set at a two‐tailed *P* value of < 0.05. To address the issue of multiple comparisons, the Benjamini–Hochberg method was used for correction when deemed necessary.

## 3 RESULTS

In the present study, a total of 107 participants were included (Figure 1), comprising 59 individuals with AD (mean age ± SD = 70.7 ± 8.0 years; 38 females), 27 with MCI (mean age ± SD = 71.1 ± 7.0 years; 13 females), and 21 CN (mean age ± SD = 70.0 ± 8.0 years; 12 females). Detailed demographic and clinical characteristics can be found in Table 1. Among them, 23 participants had a follow‐up visit at 12 months, of whom the cognitive performance at 12 months is also presented in Table 1. Out of the 23 participants, 14 were diagnosed with AD and 9 were MCI at baseline.

**TABLE 1:** Demographic and clinical characteristics of the study cohort.

	Study cohort	Longitudinal cohort Baseline	Longitudinal cohort Month 12
Demographic	*N* = 107	*N* = 23	*N* = 23
Age, y	70.7 ± 7.8	73.7 ± 6.4	/
Female, n (%)	63 (58.9%)	16 (69.5%)	/
Education, y	10.7 ± 4.5	10.6 ± 4.0	/
Cognition function			
MMSE	21.2 ± 6.6	21.4 ± 5.1	19.6 ± 6.3
AVLT‐20 min	5.5 ± 8.1	0.2 ± 0.6	0.4 ± 1.0
BNT	18.6 ± 6.7	17.7 ± 6.2	17.2 ± 6.8
STT‐A	149.2 ± 211.0	124.6 ± 60.3	169.0 ± 199.1
STT‐B	364.5 ± 322.4	445.7 ± 337.4	472.8 ± 376.7
AFT	10.9 ± 5.5	10.4 ± 5.2	9.2 ± 6.2

*Note*: Shown is mean ± standard deviation unless specified otherwise.

Abbreviations: AFT, Animal Fluency Test; AVLT, Auditory Verbal Learning Test; BNT, Boston Naming Test; IQ,R interquartile range; MMSE, Mini‐Mental State Examination; STT, Shape‐Trail Test.

Figure 2C illustrates the cortex connected with WMH at all three connectivity levels. It is important to note that the presence of WMH patterns varied, and therefore, the demographics of the samples used in the specific WMH pattern analyses are provided in Table S2 in supporting information.

### 3.1 Cortical thickness, amyloid, tau, and synaptic density in the WMH‐connected cortex

At all connectivity levels, we found that the cortical thickness and SV2A density of the WMH‐connected cortex were significantly lower compared to the WMH‐unconnected cortex. Specifically, the mean ratio of cortical thickness in the WMH‐connected cortex to the WMH‐unconnected cortex was 0.975 ± 0.035 (mean ± SD), 0.965 ± 0.049, 0.956 ± 0.064 (all *P* < 0.001) for total dWMH and 0.959 ± 0.033, 0.948 ± 0.042, 0.938 ± 0.050 (all *P* < 0.001) for total pWMH at low, medium, and high connectivity level, respectively. Similarly, the ratio of SV2A density in the WMH‐connected cortex to the WMH‐unconnected cortex was 0.944 ± 0.038, 0.933 ± 0.046, 0.933 ± 0.046 (all *P* < 0.001) for total dWMH and 0.914 ± 0.031, 0.9 ± 0.036, 0.894 ± 0.046 (all *P* < 0.001) for total pWMH at three connectivity levels.

**FIGURE 3:**
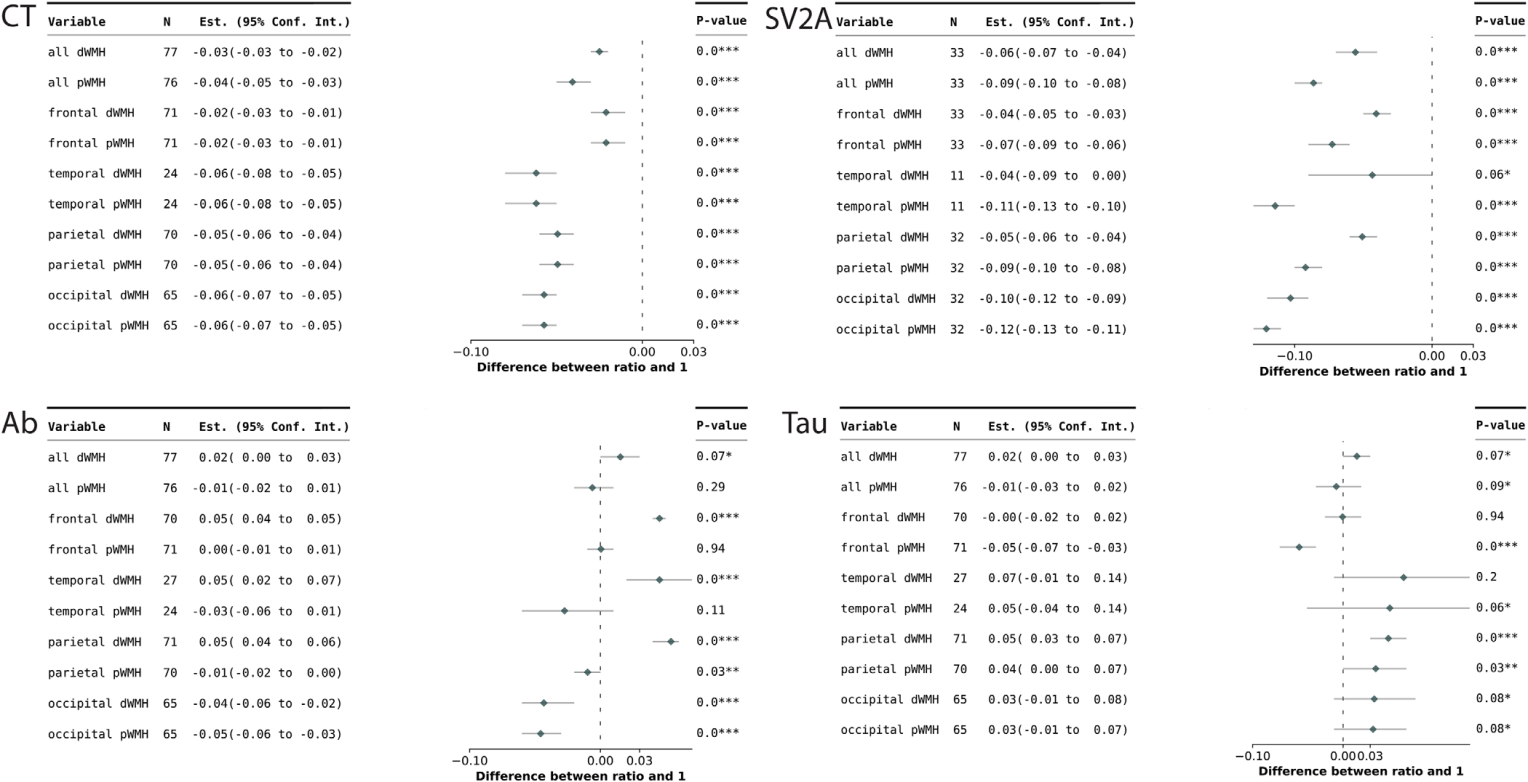
Cortical metrics in the WMH‐connected cortex versus WMH‐unconnected cortex at the low connectivity level. In the forest plots, the comparison of cortical metrics in the WMH‐connected cortex versus WMH‐unconnected cortex was calculated by subtracting one from the ratio. A value of 0 in the forest plot indicates no difference between the WMH‐connected and WMH‐unconnected cortex. Values < 0 indicate a decrease in the cortical metric in the WMH‐connected cortex compared to the WMH‐unconnected cortex, while values > 0 indicate an increase. Ab, amyloid beta; CT, cortical thickness; dWMH, deep white matter hyperintensity; pWMH, periventricular white matter hyperintensity; SV2A, synaptic vesicle glycoprotein 2A; WMH, white matter hyperintensity

In our analysis of the effect of WMH pattern, we observed marginal significance in cortical thickness comparison of the temporal dWMH‐connected/unconnected cortex at high connectivity level (ratio = 0.962 ± 0.104, *P* = 0.071). In addition, we found that the cortical thickness in all other WMH pattern–connected cortex was significantly reduced compared to the corresponding WMH‐unconnected cortex at all connectivity levels, as shown in Figure 3 and Figure S1 in supporting information.

Regarding Aβ deposition, we found that the frontal, temporal, and parietal dWMH‐connected cortex had higher levels of Aβ deposition compared to the WMH‐unconnected cortex (ratio = 1.045 ± 0.041, *P* < 0.001; ratio = 1.045 ± 0.068, *P* = 0.002; ratio = 1.054 ± 0.045, *P* < 0.001, respectively) at the low connectivity level, as well as medium and high levels. Conversely, the occipital dWMH and pWMH‐connected cortex showed lower levels of Aβ deposition at three connectivity levels (Figure 3 and Figure S1).

Additionally, we observed that the parietal dWMH‐ and pWMH‐connected cortex had higher levels of tau deposition compared to the WMH‐unconnected cortex (ratio = 1.050 ± 0.103, *P* < 0.001; ratio = 1.036 ± 0.140, *P* = 0.039, respectively) at the low connectivity level. The parietal dWMH‐connected cortex also had more tau deposition at medium and high levels. On the other hand, the frontal pWMH‐connected cortex exhibited lower levels of tau deposition at three connectivity levels.

Furthermore, except for temporal dWMH, we observed that all other WMH pattern‐connected cortex had significantly lower SV2A density compared to the WMH‐unconnected cortex at three connectivity levels.

### 3.2 Cortical thickness, amyloid, tau, and synaptic density in the WMH‐connected cortex and cognition

**FIGURE 4:**
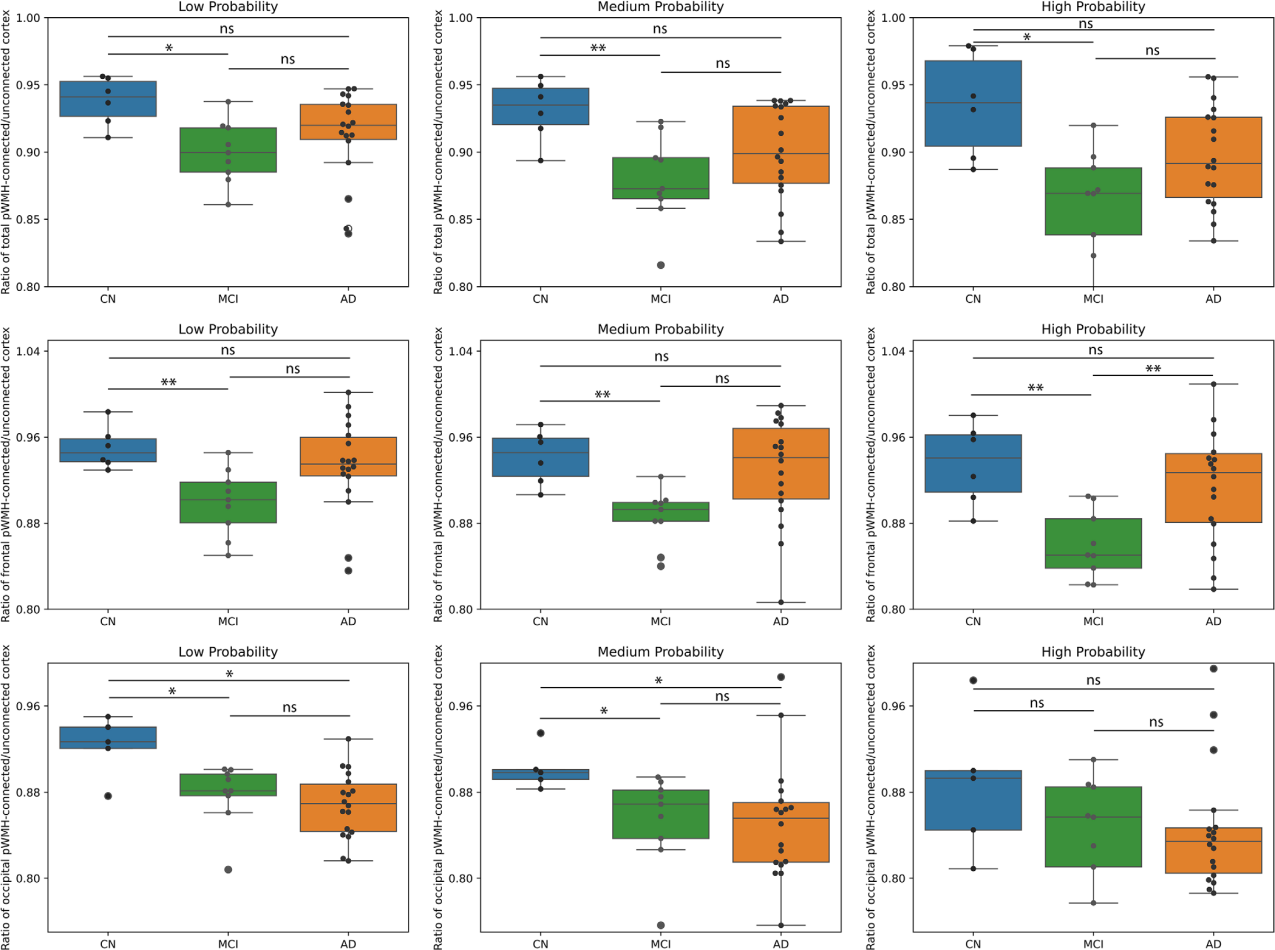
Comparison of synaptic density ratios between WMH‐connected cortex and WMH‐unconnected cortex at all connectivity levels among groups. In total and frontal pWMH‐connected cortex, synaptic density ratio was reduced in MCI compared to the CN at all connectivity levels. The MCI also had reduced ratio of synaptic density in occipital pWMH‐connected cortex at low and medium connectivity levels. *P* < 0.05; ** *P* < 0.01. AD, Alzheimer's disease; CN, cognitively normal; dWMH, deep white matter hyperintensity; MCI, mild cognitive impairment; pWMH, periventricular white matter hyperintensity; WMH, white matter hyperintensity

We found that the SV2A density ratio in total pWMH‐connected cortex was reduced in the MCI groups compared to the CN group at all connectivity levels (mean difference ± standard error [SE]: low: −0.038 ± 0.011, *P* = 0.010; medium: −0.052 ± 0.014, *P* = 0.009; high: −0.075 ± 0.022, *P* = 0.013). Similarly, the occipital pWMH‐connected cortex also showed a reduced ratio of SV2A density at low and medium connectivity levels (low: −0.045 ± 0.016, *P* = 0.049; medium: −0.048 ± 0.017, *P* = 0.039). In addition, the frontal pWMH‐connected cortex had a reduced ratio of SV2A density in the MCI group compared to the CN group at all three connectivity levels (Figure 4). We used uncorrected *P* values to increase statistical sensitivity. However, we did not find any significant difference in the ratio of cortical thickness, Aβ deposition, and tau deposition between groups at all connectivity levels (Figures S2 and S3 in supporting information).

In our study, we focused on the changes in the ratio of cortical metrics in the early stage of the AD continuum. Across the MCI group (with MMSE scores ranging from 22 to 28), we found that lower score on memory tests was significantly related to higher Aβ or tau ratio of the WMH‐connected cortex only at the low connectivity level. After adjusting for age, education years, and volume of WMH, higher Aβ ratio was significantly related to lower AVLT score in the total dWMH‐connected cortex (linear regression: estimated coefficient = −37.8, SE = 11.9, corrected *P* = 0.040) at the low connectivity level. The AVLT score was also negatively related to the ratio of tau in the total dWMH (estimated coefficient = −37.6, SE = 11.9, corrected *P* = 0.020), total pWMH (estimated coefficient = −33.1, SE = 12.5, corrected *P* = 0.038), frontal dWMH (estimated coefficient = −25.9, SE = 6.8, corrected *P* = 0.010), and frontal pWMH (estimated coefficient = −16.1, SE = 6.2, corrected *P* = 0.038)‐connected cortex.

### 3.3 Cortical thinning in the WMH‐connected cortex at follow‐up

Due to the small sample size (*n* = 23) with 1‐year follow‐up MRI and the variations in the presence of WMH in each lobe, we focused on estimating the longitudinal changes in the total dWMH‐ and pWMH‐connected cortex.

We found that the baseline dWMH‐ and pWMH‐connected cortex had reduced cortical thickness compared to the corresponding WMH‐unconnected cortex at 12 months: ratio = 0.943 and 0.925 at low connectivity, ratio = 0.920 and 0.913 at medium connectivity, ratio = 0.922 and 0.902 at high connectivity level (all *P* < 0.001).

Next, we estimated the longitudinal changes in the ratio of baseline total dWMH‐ and pWMH‐connected cortex at three connectivity levels using repeated measures ANOVA. We observed that the cortical thickness ratios of both dWMH‐ and pWMH‐connected cortex were significantly reduced within the 12‐month follow‐up period (Table 2).

**TABLE 2:** Longitudinal changes of cortical thickness ratio.

	Baseline	Month 12	Corrected *P* value^*^
Low probability			
Total dWMH	0.976 ± 0.034	0.943 ± 0.040	<0.001
Total pWMH	0.962 ± 0.020	0.925 ± 0.027	<0.001
Medium probability			
Total dWMH	0.957 ± 0.038	0.920 ± 0.050	<0.001
Total pWMH	0.954 ± 0.026	0.913 ± 0.030	<0.001
High probability			
Total dWMH	0.960 ± 0.051	0.922 ± 0.047	<0.001
Total pWMH	0.946 ± 0.033	0.902 ± 0.038	<0.001

* Compared by repeated measure tests and corrected by Benjamini–Hochberg method.

Abbreviations: dWMH, deep white matter hyperintensity; pWMH, periventricular white matter hyperintensity.

**FIGURE 5 alz13845-fig-0005:**
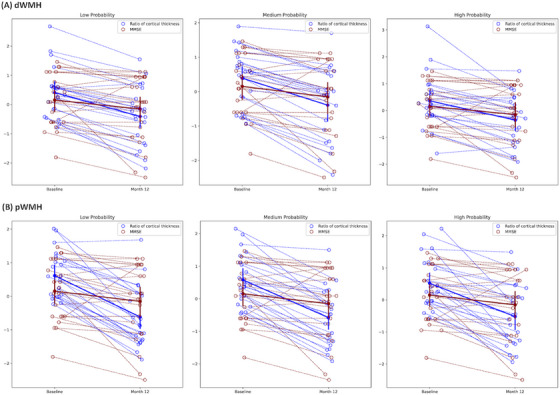
Longitudinal changes in cortical thickness ratio and MMSE at all connectivity levels. The baseline measures were standardized, and their mean and standard deviation were used to standardize the values at Month 12. The dashed lines represent individual changes in cortical thickness ratio and MMSE scores, while the solid lines represent the average changes observed in the group. dWMH, deep white matter hyperintensity; MMSE, Mini‐Mental State Examination; pWMH, periventricular white matter hyperintensity

To further investigate the relationship between changes in the ratio of WMH‐connected cortex and cognition, we used a linear mixed model with standardized preprocessing. We found that the decline in the cortical thickness ratio in the total pWMH‐ and dWMH‐connected cortex within the 12 months was faster than the decline in cognitive function measured by the MMSE at all three connectivity levels (all interaction effect *P* values < 0.05, Figure 5).

## 4 DISCUSSION

In the current study, we investigated the regional cortical abnormalities connected to WMH in AD using multimodal neuroimaging data. Our main findings can be summarized as follows: compared to WMH‐unconnected cortex, WMH‐connected cortex showed significantly lower thickness and synaptic density. In addition, higher levels of Aβ deposition in frontal, temporal, and parietal dWMH‐connected cortex, and higher levels of tau deposition in parietal WMH‐connected cortex were found, compared to their corresponding WMH‐unconnected cortex. Furthermore, the Aβ and tau ratios of WMH‐connected cortex to unconnected cortex were associated with cognitive performance in the MCI stage of the AD continuum. Last, the cortical thickness of WMH‐connected cortex was reduced more than WMH‐unconnected cortex over 12 months. These findings advance our understanding of the relationship between WMH and cortical changes in AD.

We found[Fig alz13845-fig-0005] that the cortical thickness was significantly lower in the WMH‐connected cortex compared to the WMH‐unconnected cortex in AD. Previous studies investigating SVD had similar findings. A cross‐sectional study with 213 SVD patients reported lower cortical thickness in WMH‐connected cortex compared to WMH‐unconnected cortex.^6^ Moreover, the connecting WM tracts had higher mean diffusivity values, which associated with reduced cortical thickness of WMH‐connected cortex.^6^ Another population‐based study with 930 participants showed that higher connectivity probability between WMHs and WMH‐connected cortex was related to lower cortical thickness.^5^ Several possible explanations exist for our findings. The secondary neurodegeneration of WM tracts between WMHs and WMH‐connected cortex may lead to cortical thinning in AD.^35^ In addition, WMH were reported to be associated with AD‐intrinsic processes of degeneration of cerebral GM.^36^ Our findings add to the current knowledge about the potential relationship between WMHs and regional neurodegeneration.

In addition, we found lower synaptic density of the WMH‐connected cortex compared to the WMH‐unconnected cortex. Synapse loss is one of the major pathological changes in AD,^37^ which correlates with cognitive performance.^38^ No studies have previously examined the difference in synaptic density between WMH‐connected cortex and the corresponding WMH‐unconnected cortex. The lower synaptic density, combined with the reduced cortical thickness in the WMH‐connected cortex, further indicated that the WMH may play an important role in neurodegeneration. Future studies investigating the causal relationship between WMH and neurodegeneration are warranted.

We found higher levels of Aβ deposition in the frontal, temporal, and parietal dWMH‐connected cortex, compared to their corresponding WMH‐unconnected cortex. As defined by cerebrovascular deposition of Aβ, cerebral amyloid angiopathy (CAA) is considered to play a role in the relationship between WMH and Aβ, as CAA is also associated with a high prevalence of WMHs.^39^ Future studies with susceptibility‐weighted imaging can help to examine whether WMH and Aβ deposition in cortex are linked via microbleeds and cortical siderosis. In addition, we found lower levels of Aβ deposition in the occipital WMH‐connected cortex, compared to unconnected cortex. This finding is inconsistent with previous evidence that a more posterior WMH distribution was an independent predictor of CAA,^40^ suggesting that factors other than CAA may also play a role, which warrants future investigation. Although usually considered a marker of SVD,^2^ non‐vascular mechanisms could also be involved in the WMH‐related pathophysiology in AD.^41^ It was hypothesized that part of WMHs may be secondary to AD‐related processes, such as AD‐related neurodegeneration or neuroinflammation.^41^ Many studies reported a significant relationship between WMH and amyloid markers, especially the CSF Aβ42.^3^, ^42^ The relations of amyloid load using PET with WMH have been less reported. A systematic review reported no correlation between Aβ deposition and WMH overall,^43^ while a recent study showed that amyloid load on PET correlated with a topographic pattern of WMH in non‐demented elderly, which primarily included frontal and parietal pWMH.^44^ Of note, the methods of the study (voxel‐wise multiple regression analysis using global amyloid uptake and WMH maps) were different from our current study (tractography combined with surface‐based analysis), which precluded the direct comparison of these findings. Nevertheless, our results add to the evidence base suggesting a correlation between WMHs and Aβ deposition. Potential mechanisms included that amyloid pathology may contribute to WM changes, because Aβ oligomers can exert toxicity and lead to axonal degeneration.^45^ Future studies are needed to validate these findings, and further assess the relationships between WMH and Aβ and the dynamics of these relationships.

We also found higher levels of tau deposition in parietal WMH‐connected cortex compared to the corresponding WMH‐unconnected cortex. Leys et al.^46^ reported that WM changes were more severe in the WM close to cortical areas with a high density of tau pathology. Moreover, McAleese et al.^47^ investigated the composition and etiology of parietal WM lesions in AD and found that WM legions may be associated with Wallerian degeneration that is triggered by cortical AD‐pathology including tau. Together, these findings suggested that tau pathology may play a role in the pathogenesis of WMH.^46^ The relationship between WMH and tau biomarkers was rarely found in the CSF or PET studies in the literature.^3^, ^44^ Possible explanations include that most studies assessed tau as a global biomarker, but the correlation between WMHs and tau pathology is expected to be regional or even local, instead of global.^41^ The present study investigated the link between WMHs and tau in a surface‐based manner, which enabled us to study the spatial relationships in cortical surfaces. In addition, we found that the frontal pWMH‐connected cortex exhibited lower levels of tau deposition compared to unconnected cortex, which needs to be validated in future studies. The inconsistent findings of amyloid and tau deposition across several WMH‐connected brain regions could be partially explained by the different patterns of amyloid and tau deposition in brain areas during the disease course in AD.^48^ Further studies with larger sample sizes are warranted to validate our findings.

A previous study of SVD patients showed that decreased thickness, R1, R2*, and susceptibility values in WMH‐connected cortex were related to lower scores on processing speed.^6^ However, the relations of Aβ and tau in WMH‐connected cortex to cognitive performance were less clear. We found that Aβ and tau ratios of WMH‐connected cortex to WMH‐unconnected cortex were associated with cognitive impairment in the MCI stage of the AD continuum. Our findings indicated the clinical relevance of these metrics. Future studies are needed to clarify the complex relationships among WMH, Aβ, tau, and cognition.

Previous studies investigated the effect of WMH on the remote WMH‐connected cortex using cross‐sectional data.^5^, ^6^ In addition, a study demonstrated that acute infarcts can cause cortical thinning in the lesion‐connected cortex via follow‐up MRI images.^27^ However, the effect of WMH on the thickness of WMH‐connected cortex in the long term remains to be elucidated. In the present study, we found that the thickness of both dWMH‐ and pWMH‐connected cortex decreased more than WMH‐unconnected cortex during a 12‐month follow‐up period. Our findings demonstrated that WMH could predict cortical thinning in the WMH‐connected cortex. Furthermore, we found that the decline of cortical thickness ratio in the WMH‐connected cortex was faster than the decline of cognitive function within 12 months, indicating that the changes in cortical thickness might be better and more sensitive than cognitive tests in evaluating the effect of WMH on disease progression.

The strengths of this study include comprehensive neuroimaging data and detailed cognitive assessment, with a subset of longitudinal follow‐up data. In addition, we used a dedicated protocol which combined tractography with surface‐based analysis, identifying the WMH‐connected cortex and further measuring the histopathological metrics in vivo. Moreover, we evaluated the pattern of location‐dependent effects of WMHs on cortical changes. Last, we used FreeSurfer longitudinal stream to process cortical data, which allowed us to detect the changes in cortical thickness in the submillimeter range.

We acknowledge several limitations in the present study. The sample size of the study was relatively small. The numbers of participants with SV2A PET and with follow‐up data were low. In addition, the follow‐up neuroimaging data only include change of cortical thickness. Future studies with multimodal neuroimaging data and larger sample sizes are needed to assess the change of cortex comprehensively. Considering the cortical thinning in the WMH‐connected cortex, the measures of SUVR in PET could be more vulnerable to contamination from CSF signals, partially due to partial volume effect. Nonetheless, we applied PVC methods to minimize this effect.

In conclusion, our results reveal more severe cortical thinning, Aβ and tau deposition, and synaptic density loss in the WMH‐connected cortex than in the WMH‐unconnected cortex in AD. WMHs are likely to be associated with AD‐intrinsic processes of degeneration, in addition to vascular mechanisms. Further studies are needed to disentangle the heterogeneous pathophysiological mechanisms involved in WMH sin AD.

## CONFLICT OF INTEREST STATEMENT

The authors declare no conflicts of interest. Author disclosures are available in the supporting information.

## CONSENT STATEMENT

All human subjects provided informed consent.

## Supporting information

Supporting Information

Supporting Information
