# Supplementary material for: Linking white matter hyperintensities to regional cortical thinning, amyloid deposition, and synaptic density loss in Alzheimer's disease
Source: Alzheimers Dement. 2024 Apr 22;20(6):3931–42. doi: 10.1002/alz.13845 (PMC11180938; doi:10.1002/alz.13845)
Supplement: Supplementary file 1 — Supporting Information [file ALZ-20-3931-s002.pdf]

Supplementary material

METHOD

MRI acquisition

The T1-weighted structural images were acquired with a 3-dimensional (3D) fast spoiled gradient-echo sequence, with 0.75 mm isotropic voxels (field of view read: 240 mm, output images of 360 × 439× 480 slices, repetition time (TR) / inversion time (TI) = 7.5 ms /1100 ms, echo time (TE) = 3.4 ms, flip angle = 7 degree). The 3D FLAIR acquisition parameters were: TR= 6000 ms, TE = 408 ms, and TI = 1841ms, with 0.75 mm isotropic voxels.

The parameters used for diffusion scans were very similar to those used in Human Connectome Project-Aging.[18] Diffusion images were acquired with an echo-planar imaging (EPI) sequence, and the parameters were: TR/TE= 3500/77.3 ms, voxel size = 1.5 × 1.5 × 1.5 mm, 100 slices, multiple band = 10, diffusion direction = 32/64, b = 1500/3000, phase encoding direction = PA. A pair of four b0 images with reversed phase-encode blips (AP and PA) was acquired to correct susceptibility-induced distortion.

RESULTS

Supplementary Table 1. Normality of ratios

		dWMH	pWMH	Frontal dWMH	Frontal pWMH	Temporal dWMH	Temporal pWMH	Parietal dWMH	Parietal pWMH	Occipital dWMH	Occipital pWMH
Low Probability	Cortical thickness	0.927	0.009	<0.001	<0.001	0.461	0.461	0.004	0.004	0.493	0.493
	Aβ	0.002	0.264	0.145	0.219	0.826	0.317	0.173	0.011	0.001	0.012
	Tau	0.002	<0.001	0.158	0.892	<0.001	<0.001	0.085	<0.001	<0.001	<0.001
	SV2A	0.018	0.084	0.077	0.388	0.918	0.017	0.283	0.165	0.966	0.963
Medium Probability	Cortical thickness	0.047	0.027	0.097	0.695	0.534	0.597	0.62	<0.001	0.133	0.744
	Aβ	<0.001	<0.001	<0.001	0.032	0.294	0.202	0.039	<0.001	<0.001	0.109
	Tau	0.2	<0.001	0.015	0.273	<0.001	<0.001	0.001	<0.001	<0.001	<0.001
	SV2A	0.227	0.386	0.128	0.495	0.806	0.143	0.99	0.756	0.898	0.565
High Probability	Cortical thickness	<0.001	0.104	0.007	0.587	0.163	0.509	0.081	0.001	0.028	0.726
	Aβ	<0.001	<0.001	0.073	0.018	0.229	0.015	<0.001	<0.001	0.006	0.242
	Tau	0.016	<0.001	<0.001	0.005	<0.001	<0.001	0.503	<0.001	<0.001	<0.001
	SV2A	0.009	0.409	0.853	0.338	0.931	0.499	0.108	<0.001	0.008	0.151
Longitudinal data	Cortical thickness	0.690	0.485	/	/	/	/	/	/	/	/

Shown is p-value of Shapiro-Wilk normality test. P > 0.05 represents normally distributed data while p < 0.05 represents non-normality.

Supplementary Table 2. Demographic and clinical characteristics of the sample in the specific patterns of WMH analysis

	dWMH	pWMH	Frontal dWMH	Frontal pWMH	Temporal dWMH	Temporal pWMH	Parietal dWMH	Parietal pWMH	Occipital dWMH	Occipital pWMH
	N = 77	N = 76	N = 70	N = 71	N = 27	N = 24	N = 71	N = 70	N = 65	N = 65
Age, y	70.4 ± 8.4	70.8 ± 8.0	70.7 ± 8.3	70.7 ± 8.2	71.5 ± 7.6	72.5 ± 6.2	70.6 ± 8.4	71.9 ± 7.9	70.7 ± 8.0	70.7 ± 8.0
Female	41	41	38	39	12	11	38	38	34	34
Education, y	11.0 ± 4.5	10.8 ± 4.3	11.0 ± 4.3	11.0 ± 4.3	12.4 ± 4.4	11.8 ± 4.1	10.9 ± 4.6	10.7 ± 4.5	11.1 ± 4.3	11.1 ± 4.3
Cognition function										
MMSE	21.5 ± 6.5	21.4 ± 6.5	21.1 ± 6.6	21.3 ± 6.6	23.0 ± 5.1	22.2 ± 4.9	21.0 ± 6.5	20.8 ± 6.5	21.5 ± 6.1	21.5 ± 6.1
AVLT-20min	2.5 ± 5.7	2.2 ± 5.3	1.3 ± 3.7	1.3 ± 3.7	2.4 ± 5.5	0.9 ± 1.5	1.6 ± 4.3	1.3 ± 3.5	1.0 ± 3.1	1.0 ± 3.1
BNT	18.3 ± 6.9	18.1 ± 6.8	17.8 ± 6.8	17.9 ± 6.8	18.7 ± 6.6	17.8 ± 6.4	17.7 ± 6.8	17.5 ± 6.7	17.8 ± 6.7	17.8 ± 6.7
STT-A	169.1 ± 232.9	170.9 ± 233.9	176.9 ± 241.1	175.5 ± 239.6	114.4 ± 71.1	121.8 ± 71.8	176.7 ± 241.1	178.8 ± 242.4	167.0 ± 223.5	167.0 ± 223.5
STT-B	410.1 ± 347.6	414.1 ± 348.3	425.2 ± 356.4	421.9 ± 354.8	368.9 ± 315.1	396.2 ± 323.7	420.4 ± 348.4	424.9 ± 349.1	424.8 ± 354.1	424.8 ± 354.1
AFT	10.7 ± 5.3	10.5 ± 5.1	10.1 ± 5.1	10.2 ± 5.0	10.7 ± 5.6	9.9 ± 4.9	10.3 ± 5.3	10.1 ± 5.1	10.3 ± 5.0	10.3 ± 5.0

Shown is mean ± standard deviation, unless specified otherwise. MMSE = mini-mental state examination; AVLT = auditory verbal learning test; BNT = Boston naming test; STT = shape-trail test; AFT = animal fluency test; IQR = interquartile range.

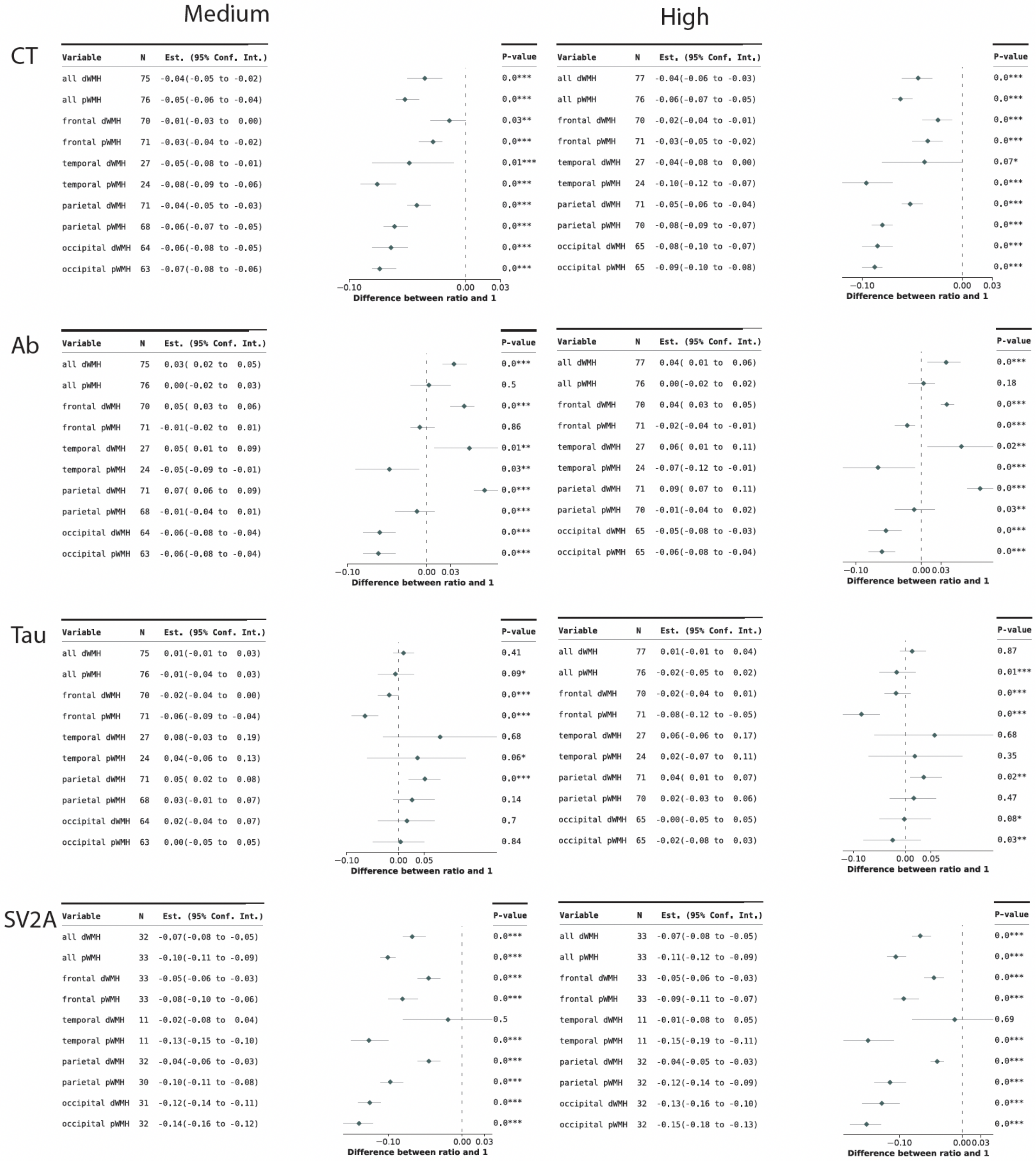

Supplementary Figure 1 Cortical metrics in the WMH-connected cortex versus WMH-unconnected cortex at median and high connectivity levels. In the forest plots, the comparison of cortical metrics in the WMH-connected cortex versus WMH-unconnected cortex was calculated by subtracting 1 from the ratio. A value of 0 in the forest plot indicates no difference between the WMH-connected and WMH-unconnected cortex. Values less than 0 indicate a decrease in the cortical metric in the WMH-connected cortex compared to the WMH-unconnected cortex, while values greater than 0 indicate an increase. dWMH = deep white matter hyperintensity. pWMH = periventricular white matter hyperintensity. CT = cortical thickness. Ab = β-amyloid. SV2A = synaptic vesicle glycoprotein 2A.

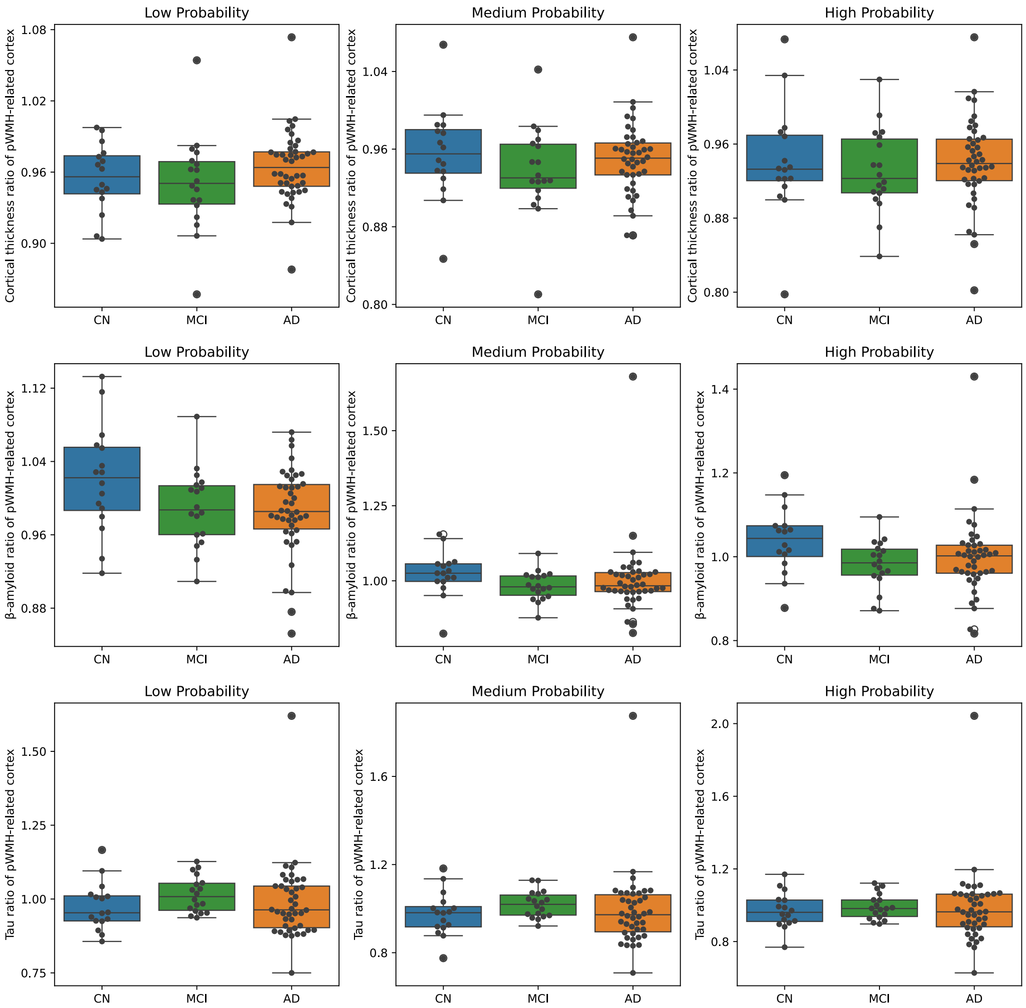

Supplementary Figure 2 Comparison of cortical thickness, β-amyloid, and tau ratios between pWMH-connected cortex and pWMH-unconnected cortex at all connectivity levels among groups. In the pWMH-connected cortex, no significant difference was found in the ratios of cortical thickness, Aβ deposition, and tau deposition among groups at all connectivity levels. pWMH = periventricular white matter hyperintensity.

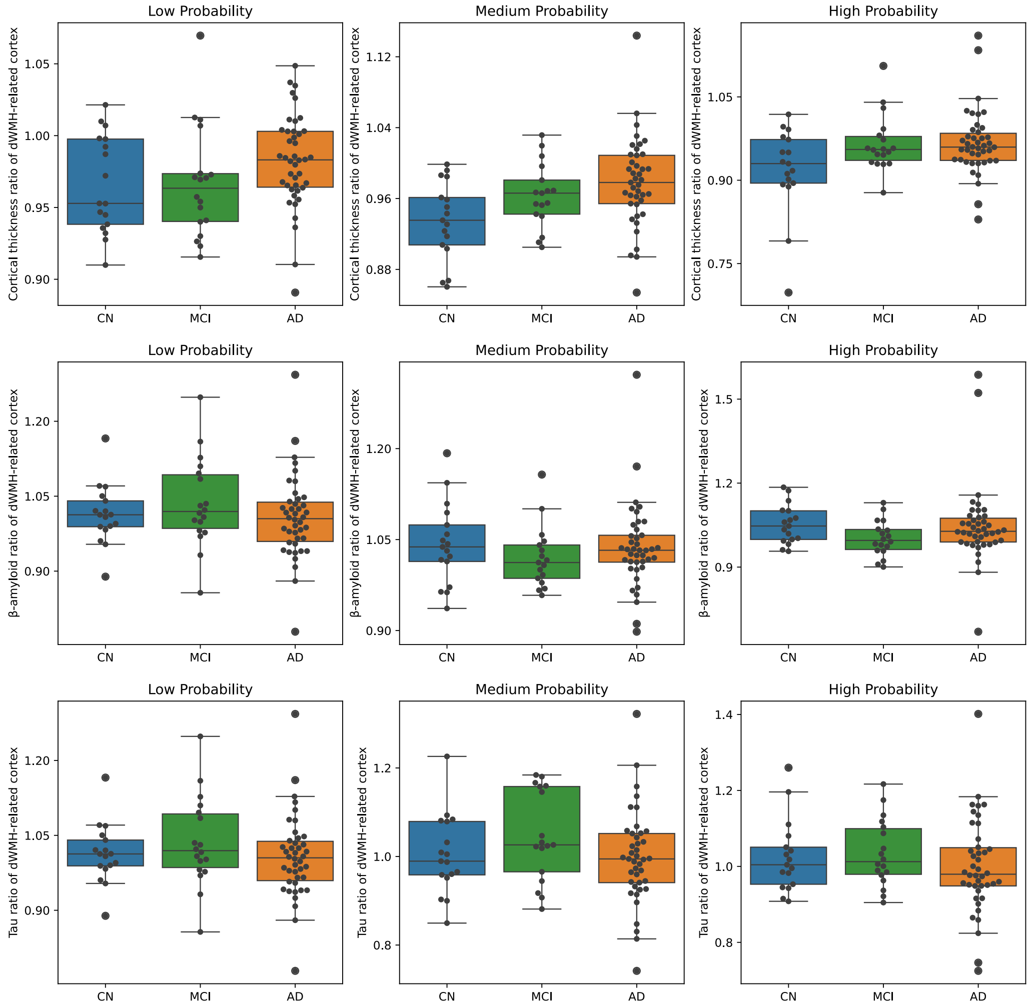

Supplementary Figure 3 Comparison of cortical thickness, β-amyloid, and tau ratios between dWMH-connected cortex and dWMH-unconnected cortex at all connectivity levels among groups. In the dWMH-connected cortex, no significant difference was found in the ratios of cortical thickness, Aβ deposition, and tau deposition among groups at all connectivity levels. dWMH = deep white matter hyperintensity.
